# Parasites contribute to ecologically dependent postmating isolation in the adaptive radiation of three-spined stickleback

**DOI:** 10.1098/rspb.2016.0691

**Published:** 2016-08-17

**Authors:** Aliya El Nagar, Andrew D. C. MacColl

**Affiliations:** School of Life Sciences, University of Nottingham, University Park, Nottingham, NG7 2RD, UK

**Keywords:** speciation, divergent evolution, local adaptation, *Gasterosteus aculeatus*, parasites

## Abstract

Spatial variation in parasitic infections is common, and has the potential to drive population divergence and the reproductive isolation of hosts. However, despite support from theory and model laboratory systems, little strong evidence has been forthcoming from the wild. Here, we show that parasites are likely to cause reproductive isolation in the adaptive radiation of three-spined stickleback. Adjacent wild populations on the Scottish island of North Uist differ greatly and consistently in the occurrence of different parasites that have substantial effects on fitness. Laboratory-reared fish are more resistant to experimental infection by parasite species from their own population. Furthermore, hybrid backcrosses between the host populations are more resistant to parasites from the parental population to which they are more closely related. These patterns provide strong evidence that parasites can cause ecological speciation, by contributing to selection against migrants and ecologically dependent postmating isolation.

## 1. Background

The ecological model of speciation, which proposes that reproductive isolation between populations occurs as a result of divergent adaptation to different environments, is widely accepted as a major explanation for how new species arise [1,2]. However, many questions still exist about how it works and the extent of its importance in comparison with other mechanisms [2,3]. One important question concerns which aspects of ecology drive the divergence between populations that initiates the process. Most effort to date has concentrated on competition and the acquisition of food, predation, and the role of the environment in mate choice [4–7]. The dynamic nature of the relationship between hosts and parasites [8], and its potential to drive divergence, suggests a role for parasites in host speciation [9–11]. While there is both theoretical underpinning for this idea [12,13] and support from model laboratory systems [14], little conclusive evidence has been forthcoming from natural populations [15]. Three prerequisites are necessary for parasites to contribute to speciation: that parasitism should differ within or between host populations, that these differences should remain consistent, and that parasites should impose fitness costs on the host [15]. These conditions are not sufficient in themselves to show that parasites do contribute to host speciation; in addition, it is necessary to document specific reproductive isolating mechanisms.

Two forms of reproductive isolation are unique predictions of the ecological speciation model [2]. Firstly, selection against immigrants, a form of premating isolation, occurs when immigrants to a population experience reduced fitness because they are not adapted to local environmental conditions [16,17]. Secondly, ecologically dependent postmating isolation occurs when hybrids between divergent populations are inferior for purely ecological reasons. Postmating isolation happens when individuals from different populations mate, but the offspring they produce are inferior in some way. This inferiority may arise because of intrinsic genetic incompatibilities between populations, or because the hybrid individuals experience an ecologically dependent mismatch to the parental environments, as a result of divergent local adaptation. Identification of these two types of postmating isolation is an important task for speciation biologists because the former can arise during several processes that lead to speciation, while the latter is exclusively a prediction of the ecological speciation model [2,18]. Separation of the two is not trivial, because reduced fitness of certain types of hybrids (notably F1s) is an expectation of both models. However, the two can be reliably separated by quantifying the performance of reciprocal backcrosses (electronic supplementary material, figure S1) in both parental environments [19]. As the different backcross types share the same degree of hybridity, the intrinsic hypothesis predicts no difference in their performance. By contrast, the ecological hypothesis predicts that backcrosses will perform best in the environments of the parental types to which they are more closely related, resulting in a switch in relative performance between environments [19]. Statistically, this switch reveals itself as an interaction between backcross type and environment when metrics of performance in the different environments are analysed [20]. Although powerful, the reciprocal backcross method has seldom been used as a research technique [20,21], perhaps because of the effort required or the difficulty of raising backcrosses between divergent populations [2]. Extension of the reciprocal backcross method to include parental types, F1s, and F2s, allows the use of line-cross analysis to explore the quantitative genetic basis of variation in (resistance) phenotypes [22].

The adaptive radiation of three-spined stickleback (*Gasterosteus aculeatus*) in waterbodies on the Scottish island of North Uist provides an ideal opportunity for testing the role of parasites in population divergence and speciation, given the variation in parasites between populations [23] and the occurrence of closely related, but reproductively isolated, sympatric ecotypes of stickleback (see below).

**Figure 1.**
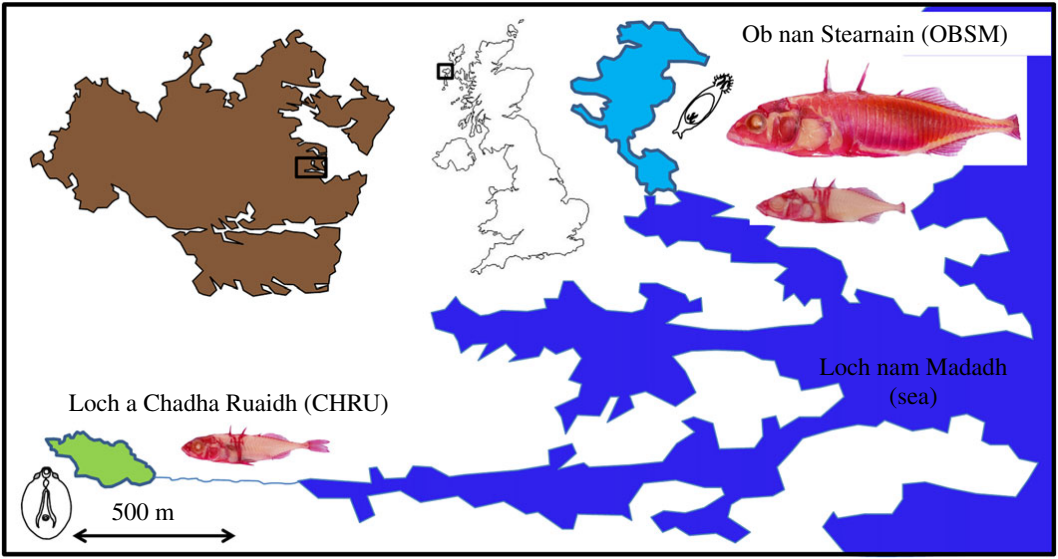
The study system, showing the relative locations of Loch a Chadha Ruaidh (CHRU, pale green) and Ob nan Stearnain (OBSM, pale blue), their location (black box) on the island of North Uist (brown), and its position in the UK. Examples of alizarin stained stickleback (caudal fins removed for genetic samples) from the two water bodies are shown. Those in the top right show a marine (top, ‘OBSM’) and a resident (‘OBSE’) fish. Line drawings of their most important parasite are shown alongside each loch: *Diplostomum* in CHRU and *Gyrodactylus* in OBSE. (Online version in colour.)

In order to infer a sufficient role of parasites in host speciation, we first show that the three necessary prerequisites for parasites to contribute to speciation are true for an adjacent pair of stickleback populations on North Uist, which we call CHRU (from a small freshwater loch) and OBSM (from a brackish lagoon) (figure 1). CHRU and OBSM exhibit substantial genetic divergence (see Material and methods). A third population (‘OBSE’) which is sympatric with, but reproductively isolated from, OBSM is morphologically similar to CHRU, demonstrating the potential for speciation in North Uist stickleback (figure 1).

We then apply the reciprocal backcross method for the first time to the question of whether parasites contribute to ecologically dependent postmating isolation, by exposing backcrosses between CHRU and OBSM to artificial infection by both a CHRU-specific parasite and an OBSM-specific parasite. We also compare the resistance of the parental types in order to add to existing work [24] suggesting that parasites could cause selection against immigrants in our system. These tests allow strong inference about the role of parasites in ecological speciation, because they arise from predictions that are unique to the model [19–21]. We show that parental types are much more resistant to the parasite that infects their own population. The resistance of backcrosses switches, depending on the parasite to which they are exposed, such that they are most resistant to the parasites from the more closely related parental population.

## 2. Material and methods

### (a) Study area

On the island of North Uist, Scotland, many isolated freshwater loch (lake) populations of stickleback show a high degree of phenotypic variation [25,26], which has probably evolved since the end of the last glaciation, approximately 16 000 years ago, before which North Uist was covered in ice [27]. Marine three-spined stickleback, from which the freshwater fish are probably derived, visit coastal brackish lagoons and some low-lying freshwater habitats to breed, but do not generally hybridize with the resident fish, which inhabit these waterbodies year round. We focus on the potential for parasites to contribute to reproductive isolation between an adjacent pair of waterbodies (figure 1), Loch a Chadha Ruaidh (CHRU, 57°36′ N; 7°12′ W), which supports a freshwater population of stickleback, and Ob nan Stearnain (OBSM, 57°36′ N; 7°10′ W), a brackish lagoon 1.5 km distant. CHRU, at an altitude of about 15 m and connected by a stream to the sea less than 400 m away, is not presently visited by marine stickleback, although the extant population was probably established from the sea, and migratory fish species (catadromous European eels, *Anguilla anguilla*) are occasionally present in the loch, so the potential for secondary contact is high. OBSM contains one of the nearest breeding populations of marine stickleback, and also supports a population of resident stickleback with freshwater morphology. We refer to the latter as saltwater residents, and give them the code OBSE. Given their phenotype (figure 1), it seems likely that this population underwent a period of evolution in freshwater before re-encountering salt water, either as a consequence of dispersal or because of rising sea levels. The sympatry of OBSE and OBSM during the breeding season, with little or no hybridization (approx. 5% hybrids, L Dean & ADC MacColl 2007–2014, unpublished data), is important in the present context, because it demonstrates that there is reproductive isolation between fish with marine and freshwater morphology following secondary contact. Values of population genetic differentiation (*F*_st_ calculated from eight putatively neutral microsatellite markers spread across the genome) suggest that CHRU fish are substantially diverged from both OBSM (0.38, *p* < 0.0001) and OBSE (0.37, *p* < 0.0001), which are much less, but still significantly, differentiated from each other (0.03, *p* < 0.0001) [28]. The apparent contradiction here, between our claim of strong reproductive isolation between OBSM and OBSE and the low *F*_st_ value arises easily when effective population sizes are large (which they are likely to be for the many interconnected populations of stickleback living in saltwater), because standard population genetics tells us that *F*_st_ is determined by the absolute number of migrants exchanged per generation [29], while reproductive isolation is defined by the proportion of hybrids [18].

### (b) Field methods

Wild fish were caught in unbaited minnow traps (Gee's, Dynamic Aqua, Vancouver) set for 24 h around the margins of waterbodies in water approximately 0.3–3 m deep. Captured fish were emptied into buckets and a haphazardly selected sample returned to the laboratory in styrofoam boxes. Fish were assayed for parasite infections within 24 h (usually within 8 h). They were first euthanized by an overdose of MS222, weighed and measured. External surfaces were carefully examined under a 10×–40× dissecting microscope, and all *Gyrodactylus* and other ectoparasites (including *Cryptocotyle* sp.) counted. Eyes were removed and dissected to count *Diplostomum* sp. and *Apatemon gracilis*. *Schistocephalus solidus* in the body cavity and *Proteocephalus filicollis* in the intestine were also recorded. All fish had their liver removed, blotted and the wet weight recorded. Hepatosomatic index (HSI), a measure of medium-term energy reserves [30], was calculated as (liver weight × 100/(fish weight − (*Schistocephalus* weight + liver weight)).

### (c) Crossing methods

Fish that were used in artificial infection experiments were raised in the laboratory from wild (grand)parents, according to standard *in vitro* methods. Briefly, testes were removed from mature wild males, minced and, in 1 ppt marine salt solution, mixed with eggs stripped from fully gravid wild females from both their own and the other population, to create pure parental and F1 crosses (electronic supplementary material, figure S1). Fertilized eggs were returned to the aquarium at the University of Nottingham, where they were reared to maturity (approx. 12–14 months). Eggs and sperm from these mature fish were then used to produce a full range of cross types (pure parentals, F1, F2, and reciprocal backcrosses) between the three populations, without inbreeding.

### (d) Experimental infections

Laboratory-raised fish, representing the full range of cross types, were exposed to infection by *Gyrodactylus* sp. and *Diplostomum* sp. in two experiments. In our experimental infections, we used parasites that were allopatric to the ones to which the fish are naturally exposed. This removes any possibility that our results are simply due to host–parasite local adaptation, making our conclusions more generic. For *Diplostomum* infections, pond snails (*Lymnaea peregra*) were collected from a lake on the campus of the University of Nottingham, returned to the laboratory and exposed to bright light in a change of water, which elicits the release of *Diplostomum* cercariae. Each fish was exposed to a dose of 20 cercariae in 1 l of aquarium water. Fish were euthanized after 48 h, their eyes removed and the total number of cercariae present was counted. For *Gyrodactylus* infections, fish were individually housed in 10 l tanks. Each fish was anaesthetized and infected with two *Gyrodactylus* worms removed from wild fish caught at Barkby Brook, Syston, Leicestershire, UK (52°42′ N; 1°04′ W). *Gyrodactylus* were then counted every fourth day until the infection was lost (which takes up to six weeks). Peak infection was used as the metric of susceptibility, although the use of other metrics of infection severity, such as number of worms at day 28 or ‘area under the infection curve’ (the sum of all counts for a fish for the whole infection period), makes no difference to the outcome of analyses. The CHRU × OBSM *Gyrodactylus* experiment used 58 fish from 26 families. The CHRU × OBSM *Diplostomum* experiment used 68 fish from 30 families.

Under UK Government Home Office regulations, which determine the treatment of animals in research, it is illegal to subject vertebrates to treatments where death is an endpoint: animals must be euthanized before this, e.g. if they begin to show symptoms which suggest the onset of severe infections. *Gyrodactylus* infections of stickleback sometimes result in secondary infection by bacteria, fungi, and protozoa. These infections develop rapidly and are commonly fatal in the absence of intervention. In our experiments, we immediately intervened in such cases by euthanizing fish, as legally required. However, we assumed that these fish would have died in the absence of intervention, and we used such cases of euthanasia to calculate ‘case fatality rates’ (CFR).

### (e) Statistical analysis

Parasite occurrence data from wild fish were analysed in Genstat 15 using generalized linear models (GLMs) with appropriate error distributions and link functions. Initially, we analysed data from artificial infection experiments using generalized linear mixed models (GLMMs) that included ‘family’ as a random term, but family never accounted for a significant proportion of the variance, and we reverted to the use of GLMs. In line-cross analyses, we followed Lynch & Walsh [22]. The Rundle–Whitlock method [19] for testing for ecologically dependent postmating isolation depends on showing that there is an interaction between backcross type and environment in determining performance [20]. Carrying out such an analysis requires the measurement of performance on the same scale in different environments. Our measures of performance are susceptibility to infection, but to two different parasites, and therefore on different scales. We therefore converted them into standardized deviates (*z* scores) [31], which can be understood as ‘environment specific susceptibility to infection’.

## 3. Results

**Figure 2.**
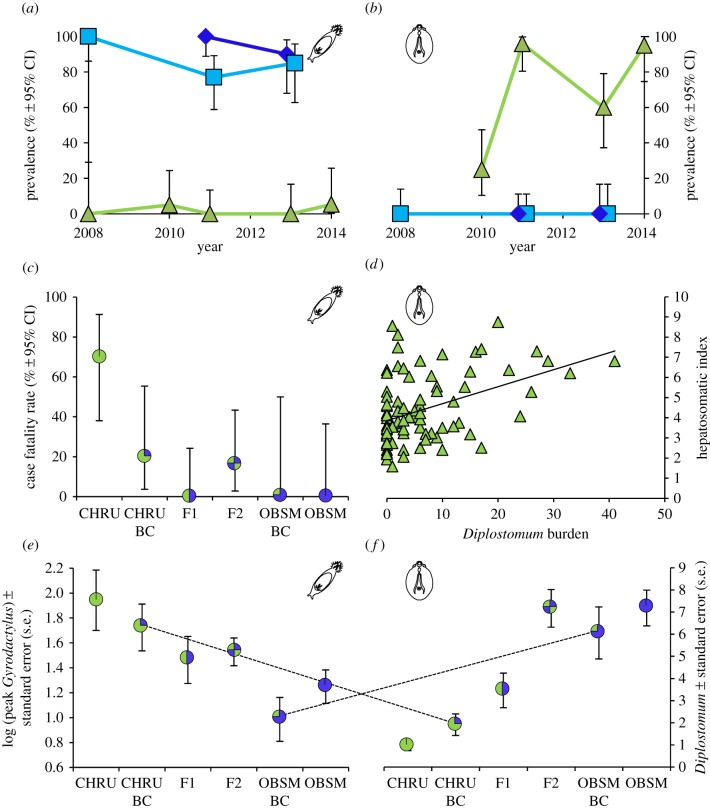
Patterns of parasitism by *Gyrodactylus* (left) and *Diplostomum* (right) on freshwater (CHRU, green triangles), marine (OBSM, dark blue diamonds), and saltwater resident (OBSE, pale blue squares) sticklebacks from North Uist. Prevalences over several years are shown in (*a*) (sample sizes are 10–30, mean 21.8) and (*b*) (sample sizes are 19–30, mean 23.1). Relationships of infection with measures of performance are shown in (*c*) and (*d*). (*c*) Case fatality rates (see Material and methods) resulting from artificial infections with *Gyrodactylus* of different cross types of CHRU (freshwater) × OBSM (marine) fish. Pie symbols illustrate the expected genomic composition of each cross type, left to right: *n* = 10, 10, 12, 13, 5, 8. (*d*) The relationship between infection with *Diplostomum* and hepatosomatic index (relative liver size), *n* = 83. Panels (*e*) and (*f*) show parasite abundances resulting from artificial infection experiments of CHRU (freshwater) × OBSM (marine) crosses. Dashed lines highlight the switch in relative performance of the two backcross types following infection with the different parasites. For *Diplostomum* left to right, *n* = 11, 12, 13, 12, 9, 11. (Online version in colour.)

Two different parasites, *Gyrodactylus* sp. (probably *arcuatus*) and *Diplostomum* sp. (probably *gasterostei*) (ectoparasitic and ocular trematodes, respectively) differed strongly in prevalence between CHRU and OBSM (figure 2*a*,*b* and table 1): *Gyrodactylus* was very common on fish in brackish water, but almost absent from the freshwater loch. By contrast, *Diplostomum* infected the majority of fish in freshwater, but was completely absent in brackish water. These patterns have remained consistent over several years, in fulfilment of the second necessary prerequisite. These differences are to some extent constrained to be true because both parasites are sensitive to changes in salinity. *Diplostomum* is not transmitted in saltwater because the freshwater snails (*Radix* spp.) that are its intermediate host are not present. *Gyrodactylus* does exist in freshwater, but is generally less prevalent and abundant than in salt (ADC MacColl 2007–2014, unpublished data). Both parasites are individual elements of larger parasite communities which differed dramatically in mean abundance between the two populations (electronic supplementary material, figure S2; table 1).

**Table 1.** Summary of statistical analyses of differences between CHRU and OBSM in prevalence and abundance of six common parasite species. Samples of at least 20 fish were taken from each population in 2011 and 2013. Population differences in prevalence were compared with *Z* tests; those for abundance, with GLMs with negative binomial errors and logarithm link functions.

parasite	statistic	d.f.	*p*-values
prevalence	*Z*		
*Gyrodactylus* sp.	9.34	1,94	<0.001
*Diplostomum* sp.	8.03	1,94	<0.001
abundance	Wald F		
*Gyrodactylus* sp.	416.31	1,94	<0.001
*Diplostomum* sp.	263.47	1,94	<0.001
*Apatemon gracilis*	130.50	1,94	<0.001
*Schistocephalus solidus*	44.30	1,94	<0.001
*Proteocephalus filicollis*	42.98	1,94	<0.001
*Cryptocotyle* sp.	129.48	1,94	<0.001

*Gyrodactylus* and *Diplostomum* have well documented effects on the fitness of fish hosts, largely through effects on survival, fulfilling the third necessary prerequisite. *Gyrodactylus* is one of the most widespread and common of all fish parasite genera. They have a direct life cycle with no need for alternative hosts, can reproduce parthenogenetically, and worms are born pregnant, giving the parasite a high potential for a rapid increase in numbers. *Gyrodactylus salaris* is well known for its disastrous effects on Norwegian salmon fisheries [32]. Previous studies of *Gyrodactylus* infections on a variety of fish species have shown that higher abundance of the parasite is associated with reduced survival of the host and other pathological effects [33–35]. In our experiments, parental CHRU and CHRU backcross fish exhibited higher CFRs following infection than other types of crosses (figure 2*c*; electronic supplementary material, table S1).

*Diplostomum* is also a widespread and common parasite of freshwater fish, with pathological effects on hosts that lead to reduced survival [36–38]. It has previously been shown that infections reduce growth rate in marine fish experimentally translocated to freshwater [24], suggesting that parasites cause selection against immigrants. Previous experimental studies have shown that infections can interfere with food intake, leading to a reduced metabolic rate and enlarged liver size [39]. In our study, *Diplostomum* infection in wild caught fish from CHRU was associated with liver enlargement (greater HSI, figure 2*d*, GLM, *F*_1,81_ = 17.86, *p* < 0.0001), suggesting metabolic consequences of infection [39]. Thus, it seems likely that both *Gyrodactylus* and *Diplostomum* reduce fitness, although this remains to be demonstrated in wild stickleback. Doing so would fulfil the necessary conditions for parasites to contribute to host speciation for CHRU and OBSM, but the results presented here are strongly suggestive.

The susceptibility of backcrosses to experimental infection clearly switched depending on the parasite to which they were exposed, with each backcross type (i.e. CHRU BC or OBSM BC) performing similarly to the most closely related parental type (figure 2*e*,*f*; GLM: environment × backcross type, *F*_1,30_ = 19.27, *p* < 0.001, backcross type *F*_1,31_ = 0.39, *p* = 0.54, environment *F*_1,31_ = 0.15, *p* = 0.71). This strongly suggests that hybrids between CHRU and OBSM, should they occur in the wild, would have reduced fitness in the environment of either parental type. We also found that the susceptibility to experimental infection by both *Gyrodactylus* and *Diplostomum* differed significantly between pure CHRU and OBSM, with fish being more susceptible to foreign parasites in both comparisons (figure 2*e*,*f*; for *Gyrodactylus*, Wald *F*_1,10_ = 9.28, *p* = 0.012; for *Diplostomum*, Wald *F*_1,20_ = 58.1, *p* < 0.001). Full quantitative genetic line-cross analysis of our data for all crosses (parentals, F1s, F2s, and backcrosses; electronic supplementary material, table S1) reveals the genetic architecture of differences between environments in host susceptibility. In *Gyrodactylus* infections, for both peak infection and CFR, differences are additive, whereas for *Diplostomum* there are also elements of dominant susceptibility. Such a pattern of dominance will also contribute to the inferiority of hybrids in the freshwater environment, further strengthening reproductive isolation.

## 4. Discussion

We have shown that parasites with known effects on fitness can differ greatly and consistently between adjacent populations. Host populations have higher, presumably evolved, resistance to the parasites in their own environment, but are susceptible to parasites that they seldom or never encounter. Resistance of backcrosses between the populations switches between environments, such that it is higher in the environment of the parental population to which each backcross type is more closely related. Fish would therefore be at a disadvantage if they migrated between populations, fuelling selection against immigrants. Crosses between the populations would also be at a disadvantage in the environments of either parental type, not just because they are hybrids *per se*, but because they are less well adapted than the parents to the ecological (parasitological) conditions existing in those environments. Thus, in the event of secondary contact between our populations, environment-specific parasites would be likely to contribute to reproductive isolation. Because neither *Gyrodactylus* sp. nor *Diplostomum* sp. is contracted through the diet, the adaptation to differences in infection is likely to be primary, and not a secondary consequence of changes in feeding ecology [15]. The total effect of environment-specific parasites on divergence and speciation in our system is likely to be greater than we have shown here, because there are at least another four parasites which are almost completely environment specific.

It has previously been shown that parasites of stickleback can have a negative effect on growth when they are translocated between locations, although previous experiments or effects have tended to be directional, rather than reciprocal [17,24]. Such effects, and those we have documented here, may be at least partly the result of differences in major histocompatibility (MHC) genes, which have been shown to be under divergent selection in stickleback [40].

In stickleback, the transition from salt- to freshwater environments is clearly associated with many ecological changes and with rapid adaptation and even speciation [41]. It is important in our study system, because both *Gyrodactylus* and *Diplostomum* are sensitive to salinity. Among the genetic changes that occur when stickleback adapt to freshwater, some of the clearest involve changes in genes that are likely to have an effect in the immune system such as TNFsf13b (BAFF), GARP, and Muc5b [42,43]. These changes could have implications for parasite resistance although the functional significance of them is unknown.

Hybrids between different taxa (commonly congeneric species) have previously been shown to have increased susceptibility to infection [44] but reciprocal exposure of (within species) host populations to (different species of) parasites from different environments does not previously seem to have been carried out. This may result from the difficulty of assembling the information to satisfy the prerequisite criteria [15] and carrying out the necessary crossing and infection experiments. We hope that our work may encourage others to see this as worthwhile, and to explore whether the contribution of spatial variation in parasitic infection to the evolution of reproductive isolation is a general one.

## Supplementary Material

electronic supplementary material

## Data accessibility

All data have been deposited in Dryad, http://dx.doi.org/10.5061/dryad.b1q15.
