# Supplementary material for: Parasites contribute to ecologically dependent postmating isolation in the adaptive radiation of three-spined stickleback
Source: Proc Biol Sci. 2016 Aug 17;283(1836):20160691. doi: 10.1098/rspb.2016.0691 (PMC5013760; doi:10.1098/rspb.2016.0691)
Supplement: electronic supplementary material [file rspb20160691supp1.docx]

**Aliya El Nagar & Andrew MacColl, Parasites contribute to ecologically dependent postmating isolation in the adaptive radiation of three-spined stickleback.**

**Electronic supplementary material**

**Supplementary Material Figure S1.** Crossing design for two adjacent Scottish three-spined stickleback populations, ‘CHRU’ and ‘OBSM’ used to generate different hybrid and pure types for experimental infections. Generation 1 fish (grandparents) were caught in the wild and crossed *in vitro*. Offspring (Generation 2) were raised in common conditions in lab aquaria. Subsequent crosses to produce generation 3 were achieved without inbreeding.

**Supplementary Material Figure S2.** Mean abundance of common parasites in CHRU (N = 45) and OBSM (N = 50). Figures shows averages (± SEM) for two years, 2011 and 2013. Note different scales on y axes. (A) *Gyrodactylus* sp. (B) *Diplostomum* sp. (C) *Apatemon gracilis* (D) *Schistocephalus solidus* (E) *Proteocephalus filicollis* (F) *Cryptocotyle* sp.

**Supplementary Material Table S1.** Quantitative genetic line cross analysis of two artificial infection experiments, involving CHRU (freshwater) x OBSM (marine) sticklebacks. Three traits were examined: (i) peak infection (maximum number of worms counted) during *Gyrodactylus* infections, (ii) ‘case fatality rate’ (whether fish had to be euthanased) during *Gyrodactylus* infections, (iii) Number of successful *Diplostomum* infections (worms counted in the eye following exposure to 20 cercariae). See main text for more details.

| Parameter | d.f. | F | P |
| --- | --- | --- | --- |
| (i) *Gyrodactylus* peak infection | 15.4% of variance |  |  |
| Additive | 1, 46 | 9.55 | 0.003 |
| Dominant | 1, 45 | 0.03 | 0.87 |
| Additive x Additive | 1, 44 | 0.59 | 0.45 |
| Dominant x Dominant | 1, 43 | 0.76 | 0.39 |
| Additive x Dominant | 1, 42 | 1.49 | 0.23 |
|  |  |  |  |
| (ii) *Gyrodactylus* case fatality rate |  |  |  |
| Additive | 1, 56 | 19.61 | <0.001 |
| Dominant | 1, 55 | 1.17 | 0.28 |
| Additive x Additive | 1, 54 | 2.18 | 0.14 |
| Dominant x Dominant | 1, 52 | 0.00 | 0.98 |
| Additive x Dominant | 1, 53 | 0.00 | 0.98 |
|  |  |  |  |
| *Diplostomum* successful infections | 46.5% of variance |  |  |
| Additive | 1, 64 | 48.00 | <0.001 |
| Dominant | 1, 64 | 11.95 | <0.001 |
| Additive x Additive | 1, 64 | 19.64 | <0.001 |
| Dominant x Dominant | 1, 63 | 3.38 | 0.07 |
| Additive x Dominant | 1, 62 | 0.04 | 0.85 |
